# Direct comparison of [^11^C] choline and [^18^F] FET PET to detect glioma infiltration: a diagnostic accuracy study in eight patients

**DOI:** 10.1186/s13550-019-0523-8

**Published:** 2019-06-28

**Authors:** Niels Verburg, Thomas Koopman, Maqsood Yaqub, Otto S. Hoekstra, Adriaan A. Lammertsma, Lothar A. Schwarte, Frederik Barkhof, Petra J. W. Pouwels, Jan J. Heimans, Jaap C. Reijneveld, Annemieke J. M. Rozemuller, William P. Vandertop, Pieter Wesseling, Ronald Boellaard, Philip C. de Witt Hamer

**Affiliations:** 1 Neurosurgical Center Amsterdam, Brain Tumour Center Amsterdam, Amsterdam UMC, location VUmc, De Boelelaan 1117, 1081 HV Amsterdam, The Netherlands; 2 Department of Radiology & Nuclear Medicine, Amsterdam UMC, location VUmc, De Boelelaan 1117, 1081 HV Amsterdam, The Netherlands; 3 Department of Anaesthesiology, Amsterdam UMC, location VUmc, De Boelelaan 1117, 1081 HV Amsterdam, The Netherlands; 4 UCL institutes of Neurology & Healthcare Engineering, Gower St, Bloomsbury, London, WC1E 6BT UK; 5 Department of Neurology, Brain Tumour Center Amsterdam, Amsterdam UMC, location VUmc, De Boelelaan 1117, 1081 HV Amsterdam, The Netherlands; 6 Department of Pathology, Brain Tumour Center Amsterdam, Amsterdam UMC, location VUmc, De Boelelaan 1117, 1081 HV Amsterdam, The Netherlands; 7 Princess Máxima Center for Paediatric Oncology, and Department of Pathology, University Medical Center Utrecht, Heidelberglaan 100, 3584 CX Utrecht, The Netherlands

**Keywords:** Glioma, [^18^F] FET, [^11^C] choline, Diagnostic accuracy, Biopsy

## Abstract

**Background:**

Positron emission tomography (PET) is increasingly used to guide local treatment in glioma. The purpose of this study was a direct comparison of two potential tracers for detecting glioma infiltration, O-(2-[^18^F]-fluoroethyl)-l-tyrosine ([^18^F] FET) and [^11^C] choline.

**Methods:**

Eight consecutive patients with newly diagnosed diffuse glioma underwent dynamic [^11^C] choline and [^18^F] FET PET scans. Preceding craniotomy, multiple stereotactic biopsies were obtained from regions inside and outside PET abnormalities. Biopsies were assessed independently for tumour presence by two neuropathologists. Imaging measurements were derived at the biopsy locations from 10 to 40 min [^11^C] choline and 20–40, 40–60 and 60–90 min [^18^F] FET intervals, as standardized uptake value (SUV) and tumour-to-brain ratio (TBR). Diagnostic accuracies of both tracers were compared using receiver operating characteristic analysis and generalized linear mixed modelling with consensus histopathological assessment as reference.

**Results:**

Of the 74 biopsies, 54 (73%) contained tumour. [^11^C] choline SUV and [^18^F] FET SUV and TBR at all intervals were higher in tumour than in normal samples. For [^18^F] FET, the diagnostic accuracy of TBR was higher than that of SUV for intervals 40–60 min (area under the curve: 0.88 versus 0.81, *p* = 0.026) and 60–90 min (0.90 versus 0.81, *p* = 0.047). The diagnostic accuracy of [^18^F] FET TBR 60–90 min was higher than that of [^11^C] choline SUV 20–40 min (0.87 versus 0.67, *p* = 0.005).

**Conclusions:**

[^18^F] FET was more accurate than [^11^C] choline for detecting glioma infiltration. Highest accuracy was found for [^18^F] FET TBR for the interval 60–90 min post-injection.

**Electronic supplementary material:**

The online version of this article (10.1186/s13550-019-0523-8) contains supplementary material, which is available to authorized users.

## Background

MRI-guided resection is the first step in multimodality treatment of diffuse gliomas [1]. The accuracy of standard T2, fluid-attenuated inversion recovery (FLAIR), and T1 contrast-enhanced weighted MRI sequences, currently used in clinical practice, [2] to detect glioma infiltration is low [3–6]. In a recent meta-analysis, the diagnostic accuracy of T1 contrast-enhanced weighted MRI sequences to identify high-glioma infiltration was lower than [11C-methyl]-methionine (^11^C-MET) PET [7]. This is in line with the Response Assessment in Neuro-Oncology (RANO) working group that recommends amino acid PET tracers to delineate glioma extent, [8] based on two studies in which ^11^C-MET and ^18^F-2-fluoro-2-deoxyglucose were directly compared [9, 10] and more indirect evidence such as extension of PET-based tumour volumes outside MRI abnormalities [11]. The most frequently used amino acid tracers are ^11^C-MET and O-(2-[18F]-fluoroethyl)-L-tyrosine ([^18^F] FET), due to its longer half-life omitting the need for an on-site cyclotron.

Choline is a well-established tracer of phospholipid metabolism and cell membrane synthesis, [12–14] although sparsely studied in untreated glioma [15–18]. Gliomas demonstrate similar uptake of the choline tracers [^11^C] choline and ^18^F-choline, [15] which is very low in normal brain compared with other tracers, potentially providing better contrast between normal brain and glioma [16, 19]*.* A dependency between choline uptake and blood-brain barrier (BBB) integrity has been described [20, 21]. On the other hand, similar relationships for tracer uptake and BBB integrity have been described for choline tracers and [^18^F] FET [22]. To the best of our knowledge, no study has directly compared a choline tracer with [^18^F] FET PET for the detection of glioma infiltration.

Therefore, we set out to compare the diagnostic accuracy of [^11^C] choline and [^18^F] FET PET in quantitative maps to detect glioma infiltration using co-registered multi-region stereotactic biopsies as reference.

## Methods

### Patients

The design of this prospective single-centre study (Amsterdam UMC, Amsterdam, the Netherlands) is described elsewhere [23]. Eight consecutive adults with a newly diagnosed supratentorial suspected diffuse glioma were included between September 2014 and March 2016. The indication for resective surgery was confirmed by the institutional multidisciplinary neuro-oncology tumour board. The eventual diagnoses proved to be two IDH1-mutated astrocytomas (WHO grade II), one IDH1-mutated 1p/19q-codeleted oligodendroglioma (grade II), one IDH1-mutated glioblastoma (grade IV) and three IDH1-wildtype glioblastomas (grade IV). Patient characteristics are presented in Table 1.

**Table 1:** Patient characteristics

Patient no.	Age (year)	Sex	Histology	WHO grade	IDH status	MGMT status	Lesion site	PET tracers	Biopsies
1	28	Female	Glioblastoma	IV	Mutant	Methylated	Left Frontal	^11^C-choline	8
2	66	Male	Glioblastoma	IV	Wildtype	Methylated	Right Frontal	^18^F-FET	8
3	37	Male	Astrocytoma	II	Mutant	Methylated	Right Frontal	Both	9
4	38	Female	Glioblastoma	IV	Mutant	Methylated	Left Frontal	Both	12
5	24	Male	Oligodendroglioma	II	Mutant	Methylated	Right Parietal	Both	8
6	21	Male	Astrocytoma	II	Mutant	Methylated	Left Temporal	Both	8
7	58	Male	Glioblastoma	IV	Wildtype	Unmethylated	Left Parietal	Both	9
8	55	Female	Glioblastoma	IV	Wildtype	Methylated	Right Parietal	Both	12

The study protocol was approved by the Medical Ethics Committee of the Amsterdam UMC, VU University Medical Centre, and registered in the Dutch National Trial Register (https://www.trialregister.nl/trial/5205, unique identifier NTR5354). Informed consent was obtained from all individual participants included in the study.

### PET protocol

Both dynamic scan protocol and pharmacokinetic modelling of [^18^F] FET have been described elsewhere [24]. Patients were required to fast for at least 4 h prior to undergoing the imaging protocol. Both [^11^C] choline and [^18^F] FET dynamic PET scans were acquired in list mode on either a Gemini TF-64 PET/CT or an Ingenuity TF PET/CT (Philips Healthcare, Best, the Netherlands), using the same scanner for each patient. Each scan started with a low-dose CT scan (30 mAs, 120 kVp) for attenuation and scatter correction purposes. Next, a 40-min dynamic scan was acquired after an intravenously injected bolus of 200 MBq [^11^C] choline. Four hours after [^11^C] choline administration, a second, 90-min dynamic scan was acquired after a bolus of 200 MBq [^18^F] FET. The list mode data were rebinned into 22 time frames for [^11^C] choline (1 × 10, 4 × 5, 2 × 10, 2 × 20, 4 × 30, 4 × 60, 1 × 150, 2 × 300, 2 × 600 s) and 22 time frames for [^18^F] FET (1 × 15, 3 × 5, 3 × 10, 4 × 60, 2 × 150, 2 × 300, 7 × 600 s). All frames were reconstructed into images with an isotropic voxel size of 2 × 2 × 2 mm^3^ using the line-of-response row-action maximum likelihood algorithm which was used for the Gemini and the “BLOB-OS-TF” algorithm for the Ingenuity. Each scan was checked and corrected for movement, if necessary, using the method described previously [24]. Maps of standardized uptake value (SUV) were normalized in activity concentrations using the injected dose per kilogram of body weight. Tumour-to-brain ratios (TBR) were calculated with a contralateral reference region, a spherical volume with a radius of 14 mm placed in the middle of the contralateral brain region. SUV and TBR were summarized for [^11^C] choline uptake between 10 and 40 min and for [^18^F] FET uptake between 20 and 40, 40–60 and 60–90 min. These intervals were chosen after visual inspection of the time-activity curves of both tracers. The reconstructions were based on static intervals for both tracers, because we demonstrated that static and dynamic parameters are quantitatively comparable in [^18^F] FET PET [24] and full kinetic analysis of choline is difficult due to the fast metabolism [25]. This resulted in two [^11^C] choline maps (SUV and TBR at 10–40 min) and six [^18^F] FET maps (SUV and TBR each at three intervals).

### MRI protocol

The MR-sequences were acquired on an Achieva 3.0 T MR-scanner (Philips), equipped with the standard head coil. Each patient was scanned with a sagittal 3D fluid attenuated inversion recovery (FLAIR) sequence (TR/TE/TI (inversion time) 4800/279/1650 ms acquired voxel size 1.12 × 1.12 × 1.12 mm, reconstructed voxel size 1.04 × 1.04 × 0.56 mm) and a sagittal 3D T1-weighted gadolinium-enhanced (T1G) sequence (TR/TE/TI/flip angle 7/3/950 ms/12°, acquired voxel size 0.98 × 0.98 × 1.00 mm, reconstructed voxel size 0.89 × 0.89 × 1.00 mm).

### Stereotactic biopsy procedure

The [^11^C] choline SUV 10–40 min, [^18^F] FET SUV 20–40 min and MRI FLAIR scan were rigidly registered to the T1G MRI (iPlan 3.0, Brainlab) and used to plan a maximum of 12 sample locations along three biopsy trajectories, avoiding vascular structures and regions related with function. Preceding the craniotomy, samples were obtained multiple regions using a previously described stereotactic procedure [26]. Biopsy sample coordinates were recorded for each imaging modality.

### Histopathology

Samples were formalin-fixed paraffin-embedded and stained using haematoxylin and eosin (HE) and Ki-67, p53 and IDH1 R132H mutation immunohistochemistry. Two expert neuropathologists independently and in consensus classified tumour presence or absence for each sample, while blinded for the imaging results, the patient’s diagnosis, and the correlations between samples. All patients had a histopathological diagnosis according to WHO 2016 criteria [27]*.*

### Statistical analysis

The index tests of the receiver operating characteristic (ROC) analysis were the intensities in the PET maps. The reference test was tumour presence in consensus between neuropathologists. Image intensities were summarized for a 1-cm^3^ region of interest (ROI), containing 125 voxels, centred at the biopsy sample coordinates (FSL, version 5.0.9, FMRIB Software Library, Analysis Group) using the 90th percentile. Missing data were omitted from analysis. Summarized intensities of each map were compared between histologically normal and tumour sample locations using two-sided Mann-Whitney *U* tests. The area under the ROC curve (AUC) with 95% confidence intervals (95% CI) and optimal cut-off with sensitivity, specificity, positive (PPV), and negative predictive values (NPV) were calculated for all maps (R package ‘pROC’, version 1.10.0). The AUCs were compared using a nonparametric analysis of clustered binary data, which corrects for the within-patient correlation of the samples [28]. Tumour presence was modelled as independent binary variable from imaging intensities by generalized linear mixed regression with logit link (R package ‘lme4’, version 1.1–13). Patient identification was included as random effect to account for within-patient correlation of the samples. Models were compared using the Akaike Information Criterion [29]. *P* values of less than 0.05 were considered significant. Subgroup analyses of high- and low-grade glioma were performed. All statistical analyses were performed using R (version 3.3.2, R Foundation). R. The study was conducted in accordance with the Standards for Reporting of Diagnostic Accuracy Studies statement (Additional file 1) [30]*.*

## Results

**Fig. 1:**
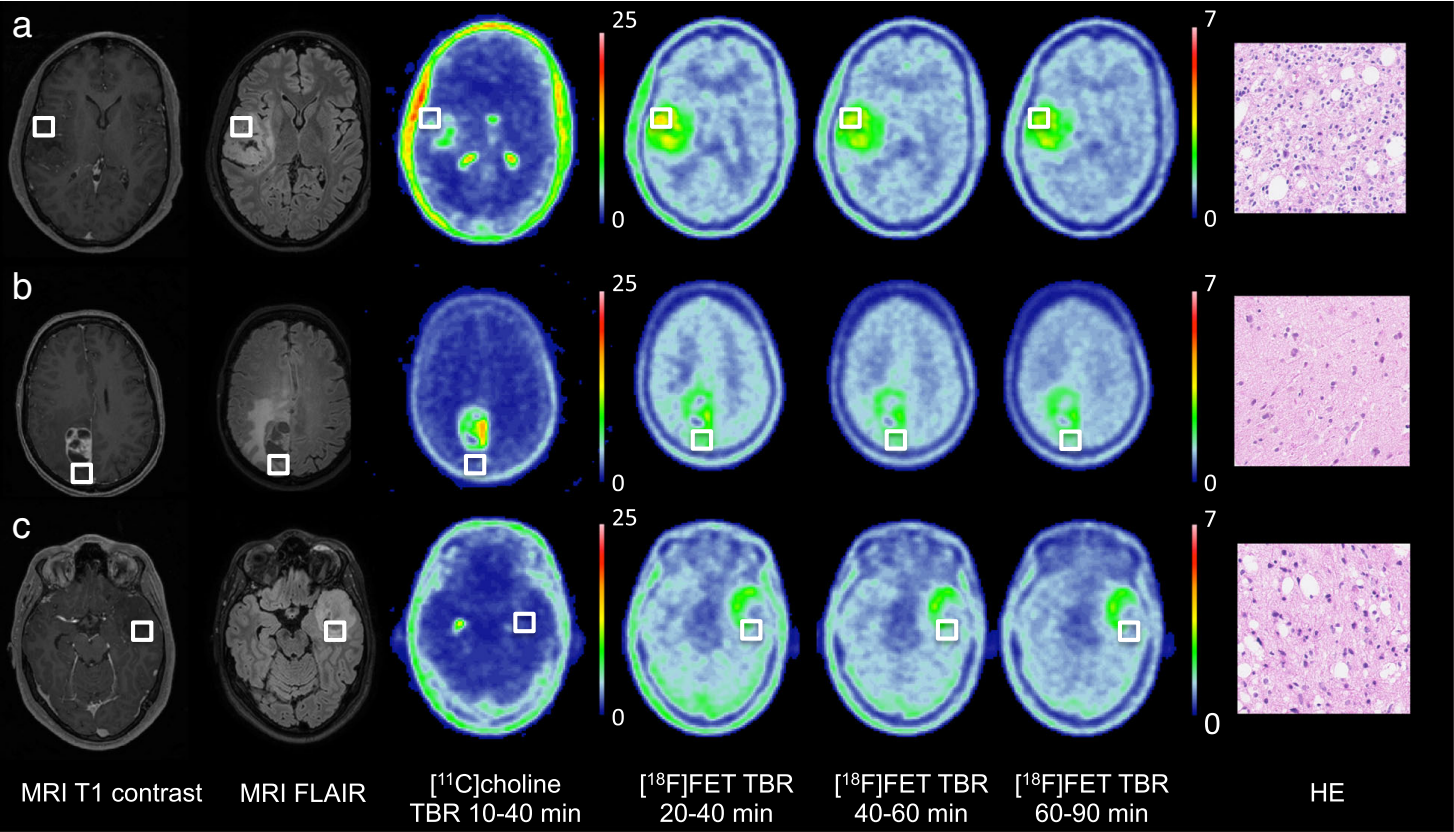
Examples of [^11^C] choline and [^18^F] FET PET scans with biopsy location (green square) and corresponding histology. **a** 24-year-old male patient with an IDH1-mutated 1p/19q-codeleted WHO grade II oligodendroglioma with a biopsy sample of clear histological tumour presence. **b** A 55-year-old female patient with an IDH1-wildtype glioblastoma with in this biopsy sample subtle histological tumour presence in the form of dispersed pleomorphic nuclei. **c** 21-year-old male patient with an IDH1-mutated grade II astrocytoma with a biopsy sample of clear histological tumour presence without visual [^11^C] choline uptake. HE = haematoxylin and eosin staining, both = [^11^C] choline and [^18^F] FET PET

Two patients with a high-grade glioma were scanned with only one tracer due to insufficient [^11^C] choline and low-quality yield of [^18^F] FET. Visual inspection showed absence of [^11^C] choline uptake in patients three and six (Fig. 1c), both with an IDH1-mutated astrocytoma (WHO grade II). All patients displayed clear [^18^F] FET uptake. Median time between PET scan and surgery was 6.5 days (range 2–12).

A total of 74 biopsy samples were acquired, with a median of 8.5 samples (range 8–12) per patient of which 54 (73%) were classified as tumour and 20 (27%) as normal. In the 49 samples of high-grade gliomas, 32 (65%) were classified as tumour and 17 (35%) as normal. In the 25 samples of low-grade gliomas, 22 (88%) were classified as tumour and 3 (12%) as normal. Of the 66 samples with [^11^C] choline data, 50 (76%) were classified as tumour and 16 (24%) as normal. In the 41 samples of high-grade gliomas, 28 (68%) were classified as tumour and 13 (32%) as normal. Of the 66 samples with [^18^F] FET data, 49 (74%) were classified as tumour and 17 (26%) as normal. In the 41 samples high-grade gliomas, 27 (66%) were classified as tumour and 14 (34%) as normal. Representative examples of the imaging and histology are shown in Fig. 1. No biopsy-related complications occurred.

### Comparison of [^11^C] choline PET standardized uptake values and tumour-to-brain ratios

**Fig. 2:**
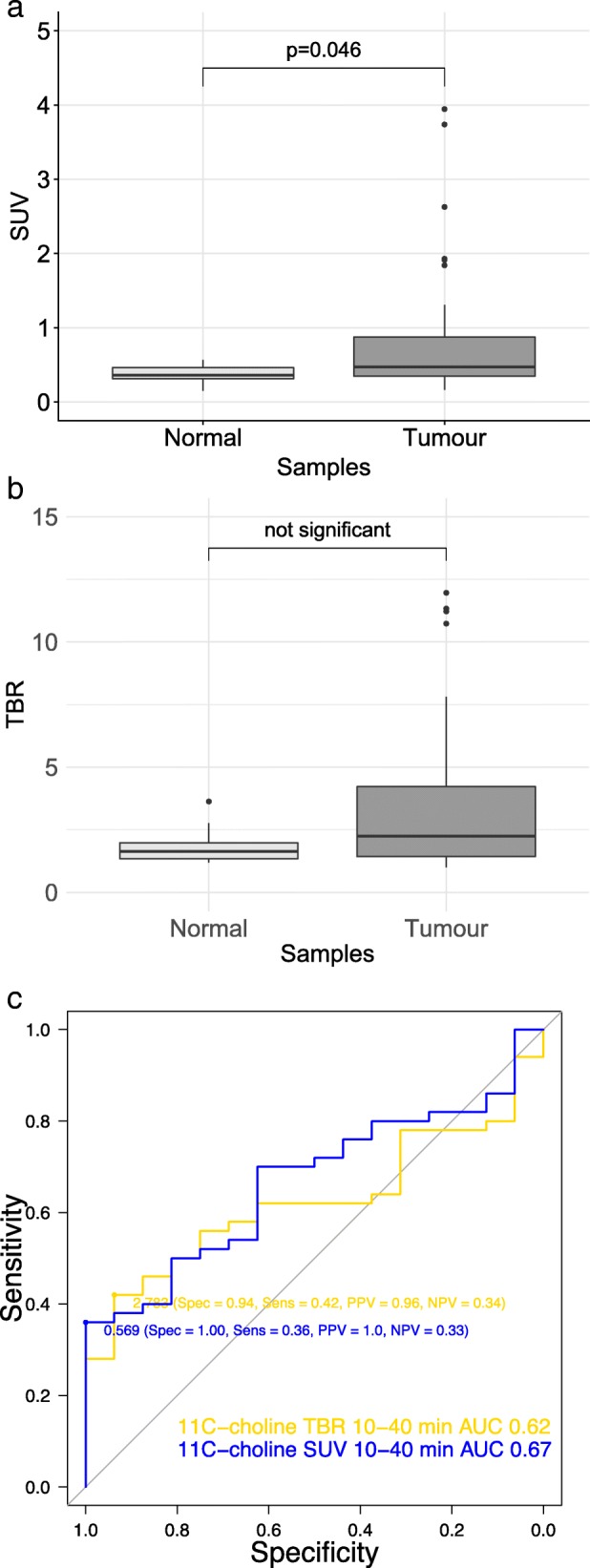
Comparison of [^18^F] FET PET maps: **a** Boxplot of [^18^F] FET standardized uptake values and tumour-to-brain ratios at 20–40, 40–60 and 60–90 min for normal (light grey) and tumour (dark grey) samples. Receiver operating characteristics curves for **b** standardized uptake values (dotted line) and **c** tumour-to-brain ratios (line) at 20–40 min (blue), 40–60 min (red) and 60–90 min (yellow) to detect tumour presence

SUV was significantly higher in tumour samples than in normal samples, and no difference was observed for TBR between tumour samples and normal samples (Fig. 2a). In high-grade gliomas, both SUV and TBR were significantly higher in tumour samples than in normal samples (Additional file 2A). In low-grade gliomas, no difference was observed for SUV and TBR between tumour and normal samples (Additional file 3A). The diagnostic accuracy for SUV and TBR for [^11^C] choline PET measurements to detect tumour presence was similar (AUC (95% CI): 0.67 (0.51–0.83) versus 0.63 (0.37–0.88), not significant) (Fig. 2b). In high-grade gliomas, diagnostic accuracy of SUV and TBR were similar (0.76 (0.56–0.96) versus 0.73 (0.47–1.00), not significant) (Additional file 2B). In low-grade gliomas, diagnostic accuracy of SUV was higher than that of TBR (0.77 (0.39–1.00) versus 0.61 (0.27–0.94), *p* < 0.001) (Additional file 3B). Based on the significant difference in uptake between tumour and normal samples, we used [^11^C] choline PET SUV for further analyses to compare with [^18^F] FET.

### Comparison of [^18^F] FET PET standardized uptake values and tumour-to-brain ratios at 20–40, 40–60 and 60–90 min

**Fig. 3:**
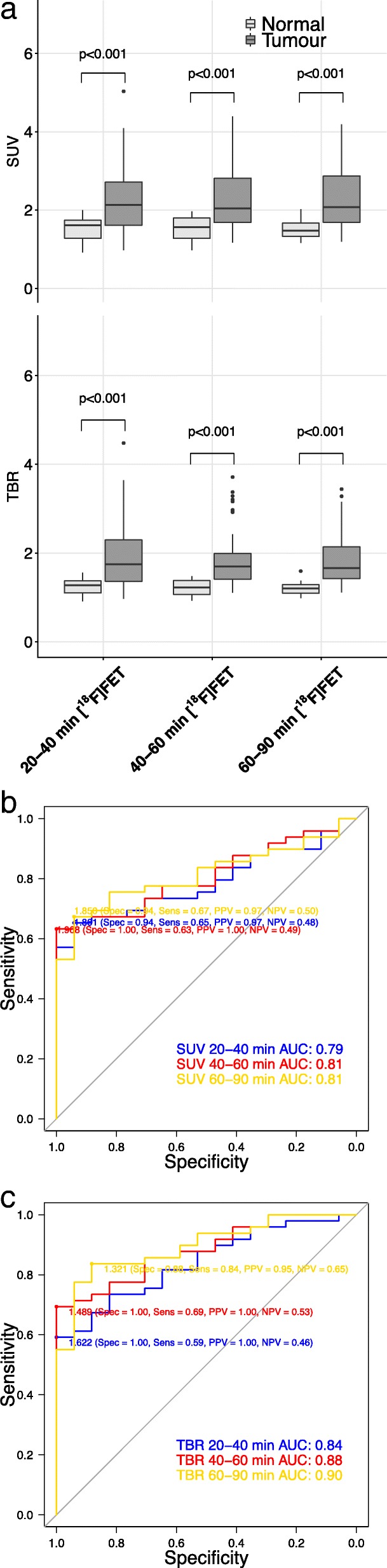
Comparison of [^18^F] FET PET maps: **a** Boxplot of [^18^F] FET standardized uptake values and tumour-to-brain ratios at 20–40, 40–60 and 60–90 min for normal (light grey) and tumour (dark grey) samples. **b** Receiver operating characteristics curves for standardized uptake values (dotted line) and **c** tumour-to-brain ratios (line) at 20–40 min (blue), 40–60 min (red) and 60–90 min (yellow) to detect tumour presence

The SUV and TBR of all intervals were higher in tumour samples compared in normal samples in all gliomas (Fig. 3a) and high-grade gliomas (Additional file 4A). In low-grade gliomas, there was no difference between tumour and normal samples’ SUV and TBR of all intervals (Additional file 5A). The 60–90-min TBR diagnostic accuracy was the highest and significantly higher than all SUVs (AUCs in Table 2 and ROC curves in Fig. 3b, c). In high-grade gliomas, the diagnostic accuracy was highest in the 40–60 and 60–90 min in TBR, with a significantly higher accuracy of 40–60 min TBR than 20–40 min SUV (Additional file 4B). In low-grade gliomas, the 40–60 min TBR diagnostic accuracy was the highest and significantly higher than 40–60 min and 60–90 min SUV (Additional file 5B). The TBR of [^18^F] FET at 60–90 min was used for further analyses to compare with [^11^C] choline, because of the higher diagnostic accuracy.

**Table 2:** Comparison of diagnostic accuracy of [^18^F] FET SUV and TBR intervals in 7 patients with 66 samples

				SUV		TBR		
				20–40 min	40–60 min	60–90 min	20–40 min	40–60 min
		AUC		0.79	0.81	0.81	0.84	0.88
			95%CI	0.59–0.99	0.63–0.99	0.64–0.99	0.70–0.98	0.76–1.00
SUV	40–60 min	0.81	0.63–0.99	*p* = 0.377				
	60–90 min	0.81	0.64–0.99	*p* = 0.478	*p* = 0.747			
TBR	20–40 min	0.84	0.70–0.98	*p* = 0.166	*p* = 0.466	*p* = 0.595		
	40–60 min	0.88	0.76–1.00	*p* = 0.026	*p* = 0.026	*p* = 0.043	*p* = 0.158	
	60–90 min	0.90	0.79–1.00	*p* = 0.033	*p* = 0.043	*p* = 0.047	*p* = 0.082	*p* = 0.355

### Comparison of [^11^C] choline and [^18^F] FET PET

**Fig. 4:**
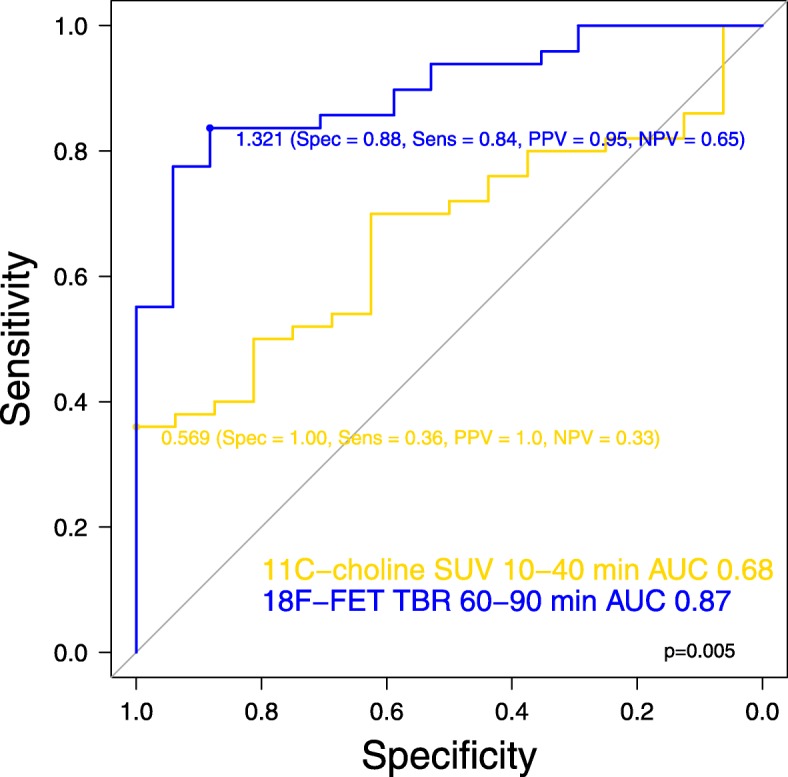
Receiver operating characteristic curve of [^11^C] choline standardized uptake values at 10–40 min (yellow) and [^18^F] FET tumour-to-brain ratios at 60–90 min (blue) (*n* = 6)

The diagnostic accuracy to detect tumour of the best quantitative map using [^18^F] FET is higher than the best quantitative map using [^11^C] choline (AUC (95% CI): 0.87 (0.75–1.0) and 0.68 (0.51–0.85), *p* = 0.005), as plotted in Fig. 4. This was similar in high-grade gliomas, although not significant, while the diagnostic accuracy in low-grade gliomas was comparable between [^18^F] FET and [^11^C] choline (Additional file 6). The TBR of [^18^F] FET PET at 60–90 min was strongly associated with tumour presence in multivariable models, but [^11^C] choline was not (Table 3). In high-grade gliomas, both tracers were associated with tumour presence, while in low-grade gliomas none (Additional file 7).

**Table 3:** Multivariable regression analysis with tumour-to-brain ratios of [^11^C] choline and [^18^F] FET PET (*n* = 6)

	Coefficient	Standard Error	*P* value	AIC model
(Intercept)	− 15.908	6.826	0.020	41.5
[^11^C] choline SUV 10–40 min	7.536	6.492	0.246	
[^18^F] FET TBR 60–90 min	10.216	4.398	0.020	

## Discussion

Our study demonstrates that [^18^F] FET PET is more accurate than [^11^C] choline PET to detect glioma infiltration. Furthermore, our results suggest that the [^18^F] FET PET 60–90-min interval might have a higher diagnostic accuracy than the 20–40-min interval.

Few studies have compared [^18^F] FET and [^11^C] choline tracers in glioma [16, 18, 31]. These studies did not address glioma infiltration in patients, but differentiation of radiation necrosis and glioma recurrence in animals, [31] detection of metabolic hotspots for grading in low- and high-grade glioma [16] and the use of [^11^C] choline PET in [^18^F] FET-negative low-grade gliomas [18]. In these studies, [^18^F] FET PET was better than [^11^C] choline PET.

Our findings support the debate on the best interval for [^18^F] FET PET favouring the longer interval of 60–90 min over the recommended 20–40-min interval [32]. Others have found better detection of diffuse glioma at intervals over 60 min compared to shorter intervals as well [33]. On inspection of our PET maps, this can be explained by improved contrast between tumour and normal brain due to the mitigation of uptake in surrounding brain tissue. It remains to be determined whether the modest increase in accuracy of longer scan intervals is set off by the longer procedure time between tracer injection and scan completion.

Our findings of the accuracy of [^18^F] FET PET to discern tumour from normal confirm that of others. In a recent meta-analysis, pooling of seven [^18^F] FET PET studies resulted in an accuracy of 0.89 [6]. Combining MRI and FET PET was more accurate than MRI alone, [34] and [^18^F] FET PET accuracy was higher than intra-operative 5-ALA fluoresence [35]*.* The [^18^F] FET tracer seems to perform similar to the ^11^C-MET tracer [7]. Of interest, the patient with a WHO grade II oligodendroglioma had higher uptake of both [^18^F] FET and [^11^C] choline than the WHO grade II astrocytomas. This may be attributable to the higher proliferation and microvessel counts in oligodendrogliomas [16, 36, 37]. *The lower accuracy in low-grade compared to high-grade gliomas has been described before* [38]*.*

The profound difference in [^18^F] FET and [^11^C] choline uptake in glioma may have several explanations. First, the cellular transport mechanism differs between these tracers. Uptake of [^18^F] FET is mediated by system l amino acid transporters (LAT) and uptake of [^11^C] choline correlates with choline transporter-like 1 (CTL-1) expression [39, 40]. Second, choline metabolism is very fast, with the parent fraction of the tracer decreasing in 15 min to 27%, [41] compared to 87% in 120 min for [^18^F] FET [42], resulting in a better tracer availability of [^18^F] FET. Finally, the dependency of [^18^F] FET uptake on breakdown of the BBB was less than that of [^11^C] choline, with high [^18^F] FET uptake also in tumour regions outside the area of contrast enhancement (Additional file 8). This is in line with preclinical studies and one human study comparing amino acid and choline tracers for the differentiation of glioma recurrence and radiation necrosis [21, 31, 43]. Other preclinical studies, however, found similar and even higher BBB dependency of [^18^F] FET compared with choline tracers [19, 22]. A potential explanation is the use of an acute radiation injury model in these studies, which has a more profound inflammatory response and more BBB disruption than seen in radiation necrosis.

A practical implication from our study is that glioma resections and radiation oncology plans may consider use of [^18^F] FET PET at late intervals to include glioma infiltration in local treatment plans. Amino acid tracers have been recommended to guide glioma resections [8]*.*

Our study has some limitations. The number of patients for our detailed imaging protocol, which can be demanding for patients, is necessarily limited, although the number of samples is relatively large. The assessment of tumour presence by neuropathologists as a reference test is known to be subject to interobserver variation [44], which is partly accounted for by consensus assessment.

## Conclusion

The [^18^F] FET tracer is more accurate than [^11^C] choline to detect glioma infiltration. The most accurate [^18^F] FET maps are based on static TBR for the interval 60–90-min post-injection.

## Additional files

Additional file 1: Standards for Reporting of Diagnostic Accuracy Studies statement checklist. (DOCX 40 kb)

Additional file 2: Comparison of [^11^C] choline SUV and TBR in high-grade gliomas. (PDF 94 kb)

Additional file 3: Comparison of [^11^C] choline SUV and TBR in low-grade gliomas. (PDF 96 kb)

Additional file 4: Comparison of [^18^F] FET SUV and TBR in high-grade gliomas. (PDF 111 kb)

Additional file 5: Comparison of [^18^F] FET SUV and TBR in low-grade gliomas. (PDF 108 kb)

Additional file 6: ROC curve of [^11^C] choline and [^18^F] FET in high- and low-grade gliomas. (PDF 66 kb)

Additional file 7: Multivariable regression analysis with tumour-to-brain ratios of 11C-choline and 18F-FET PET for high- and low-grade gliomas. (XLSX 38 kb)

Additional file 8: Boxplot of [^11^C] choline and [^18^F] FET SUV in samples with and without contrast enhancement in enhancing gliomas. (PDF 5 kb)

## Abbreviations

[^18^F] FET: O-(2-[^18^F]-fluoroethyl)-l-tyrosine
95% CI: 95% confidence intervals
AUC: Area under the ROC curve
BBB: Blood-brain barrier
FLAIR: Fluid-attenuated inversion recovery
HE: Haematoxylin and eosin
PET: Positron emission tomography
RANO: Response Assessment in Neuro-Oncology
ROC: Receiver operating characteristic
ROI: Region of interest
SUV: Standardized uptake value
T1G: T1-weighted gadolinium-enhanced
TBR: Tumour-to-brain ratio
