# Supplementary figures and images for: Direct comparison of [11C] choline and [18F] FET PET to detect glioma infiltration: a diagnostic accuracy study in eight patients
Source: EJNMMI Res. 2019 Jun 28;9:57. doi: 10.1186/s13550-019-0523-8 (PMC6598977; doi:10.1186/s13550-019-0523-8)

Online Resource 2:  
Comparison of [11C]choline SUV and TBR in high-grade gliomas

A)

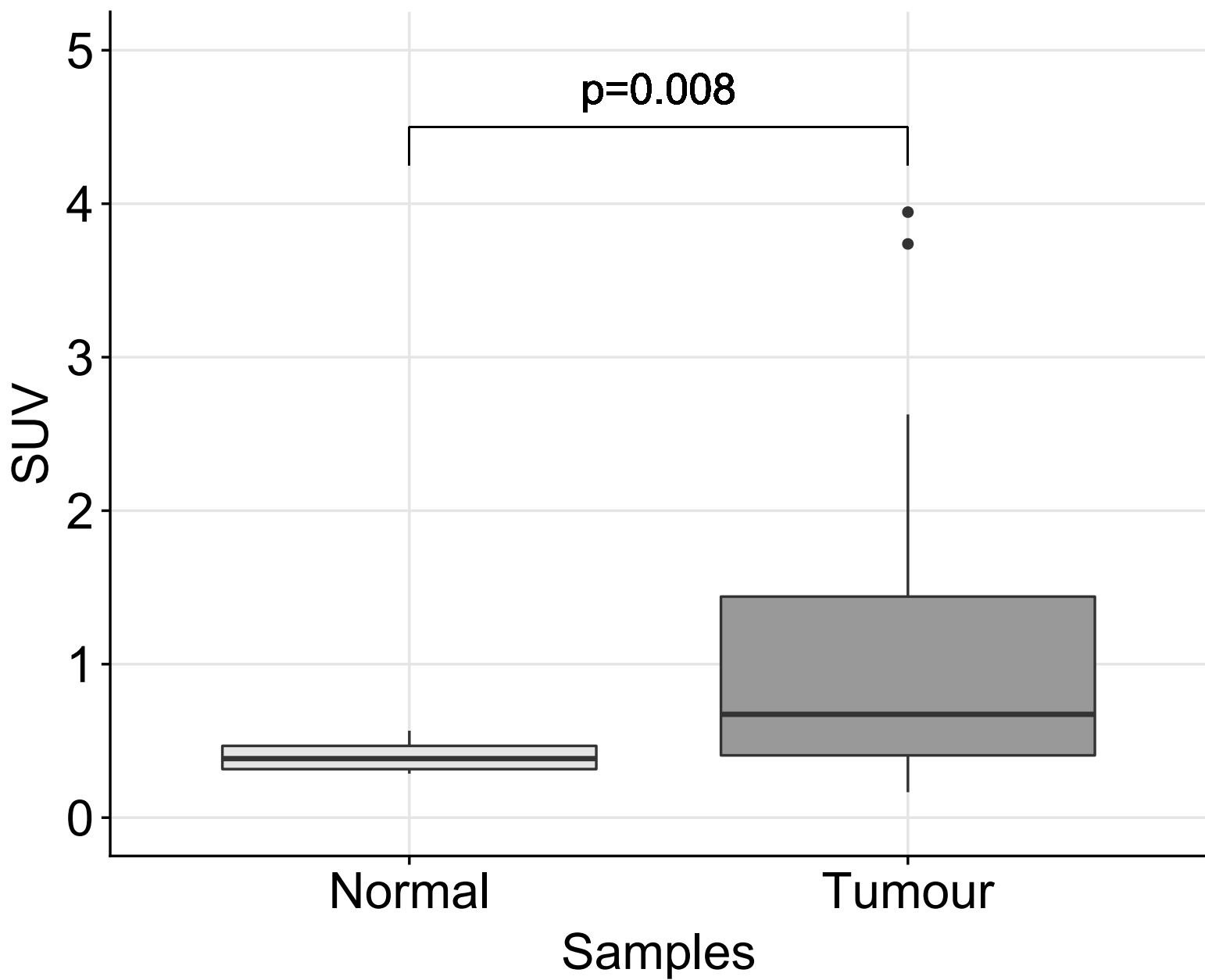

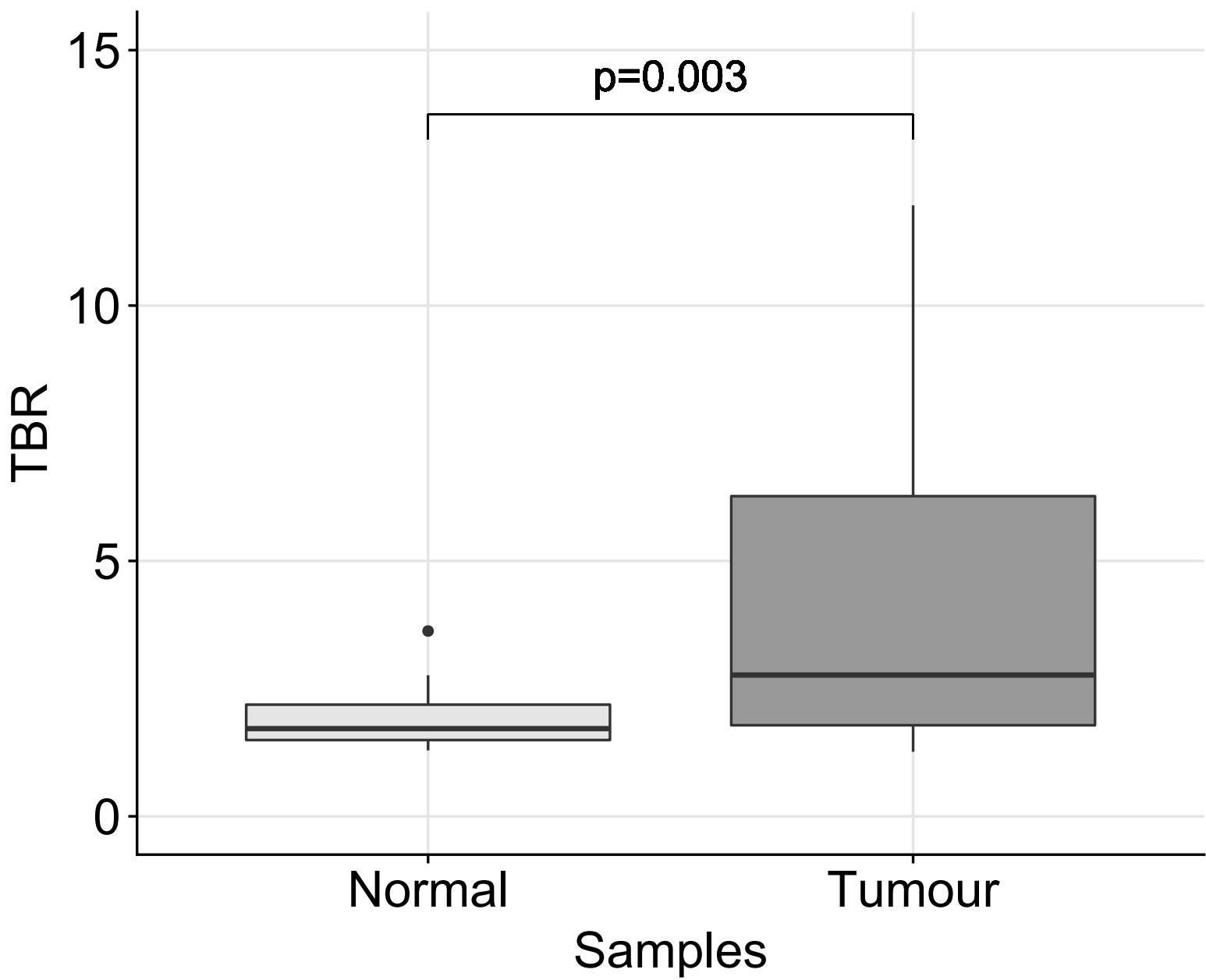

B)

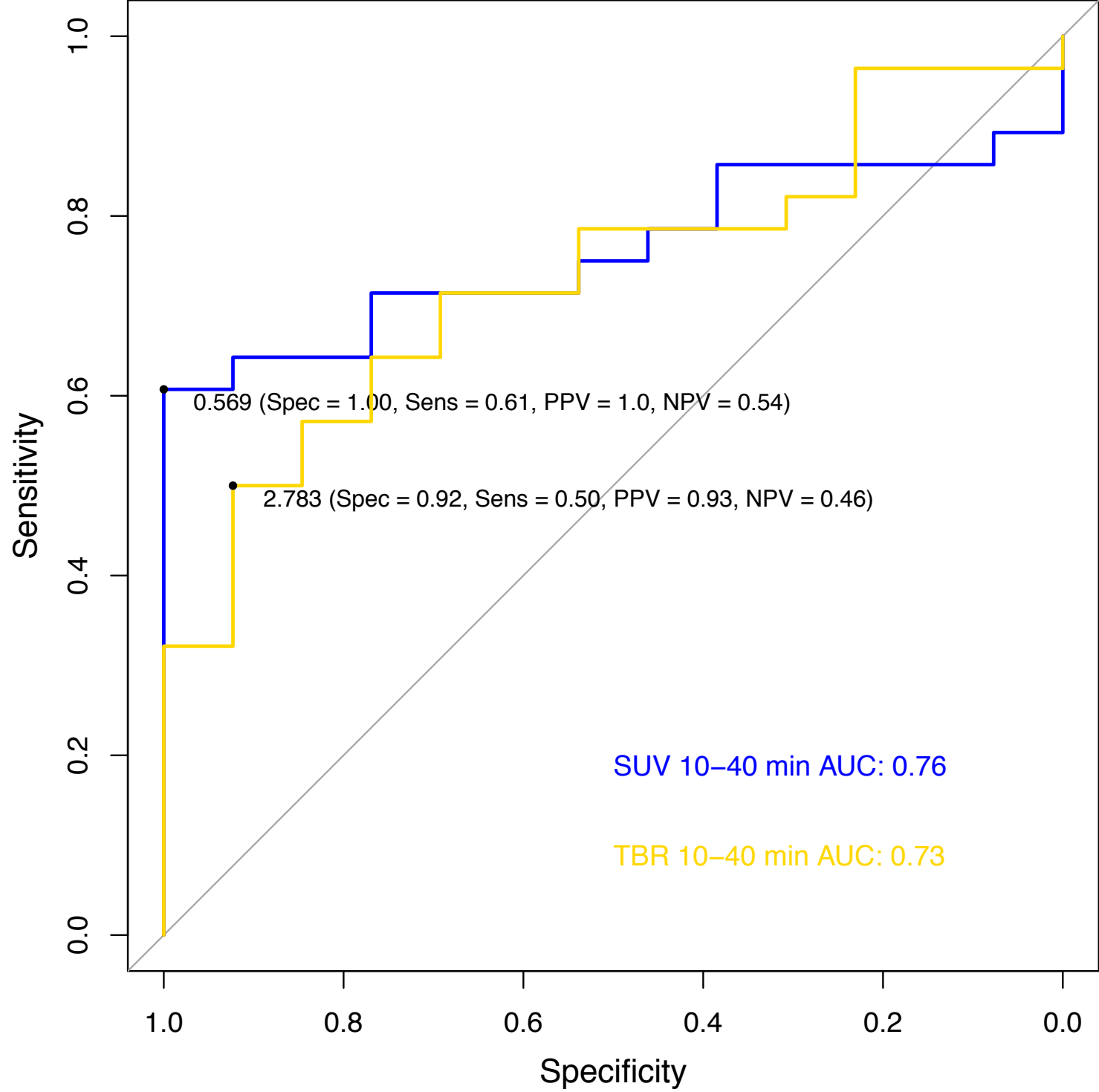

Supplement: Supplementary file 2 — Comparison of [11C] choline SUV and TBR in high-grade gliomas. (PDF 94 kb) [file 13550_2019_523_MOESM2_ESM.pdf]

Online Resource 3:  
Comparison of [11C]choline SUV and TBR in low-grade gliomas

A)

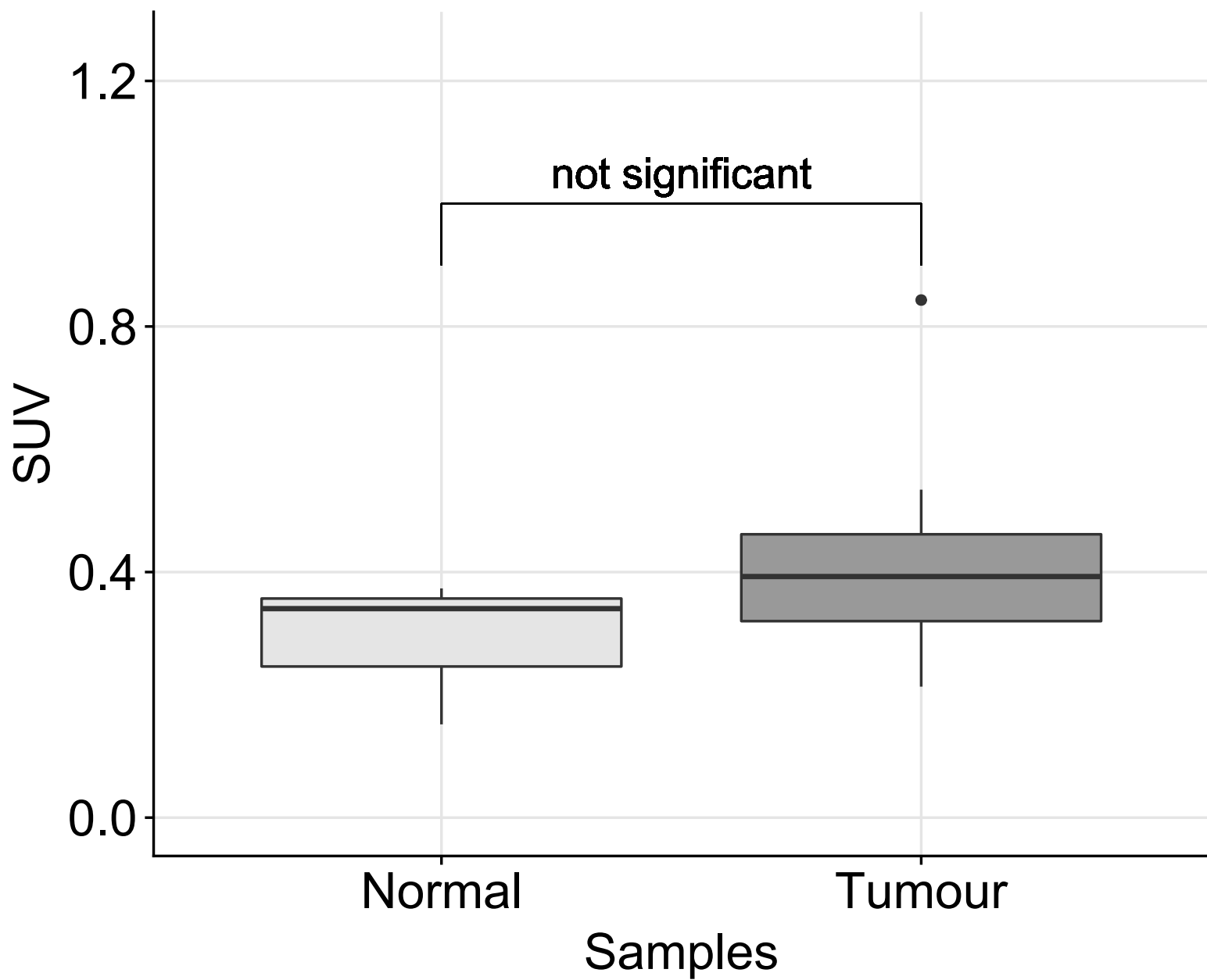

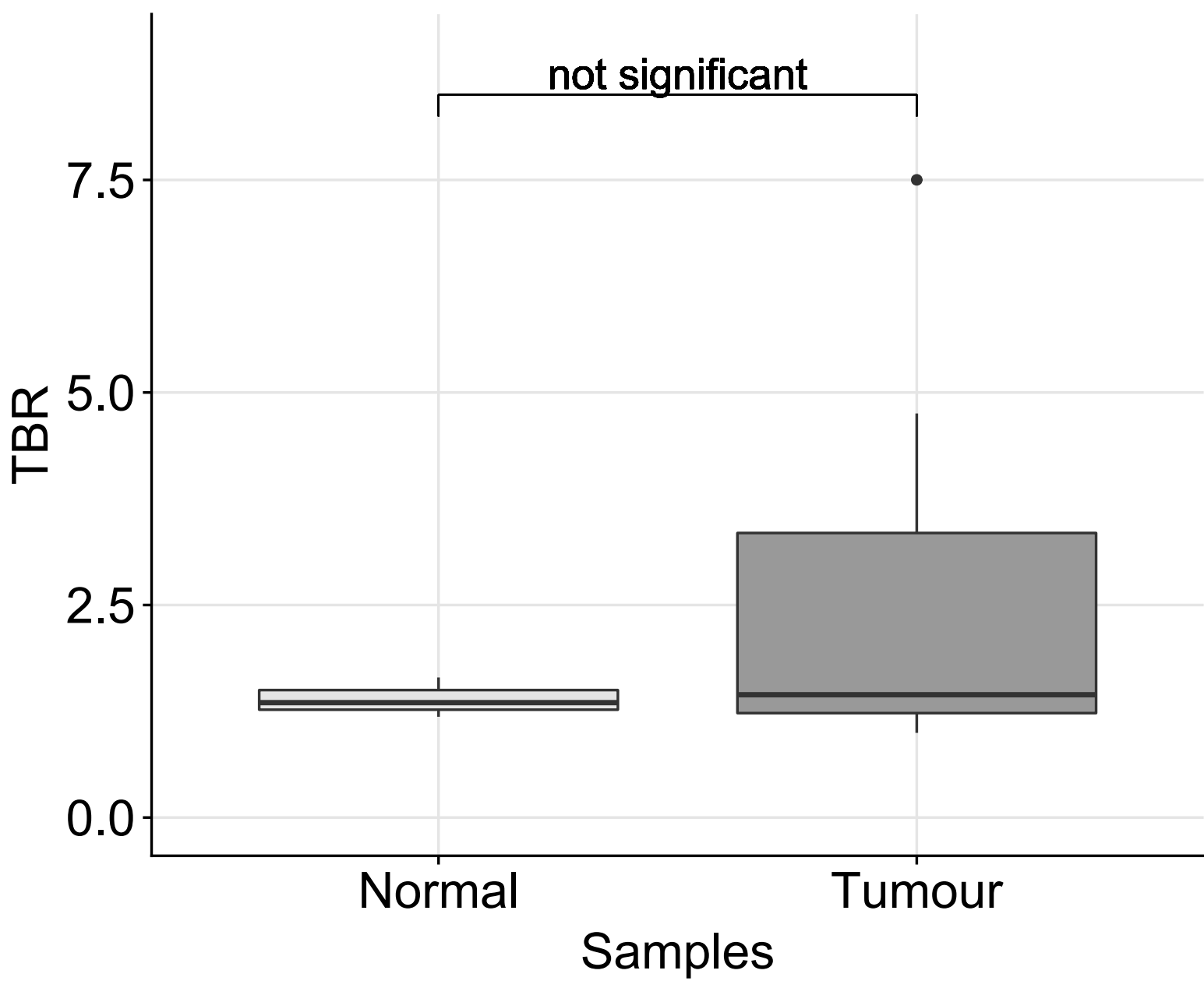

B)

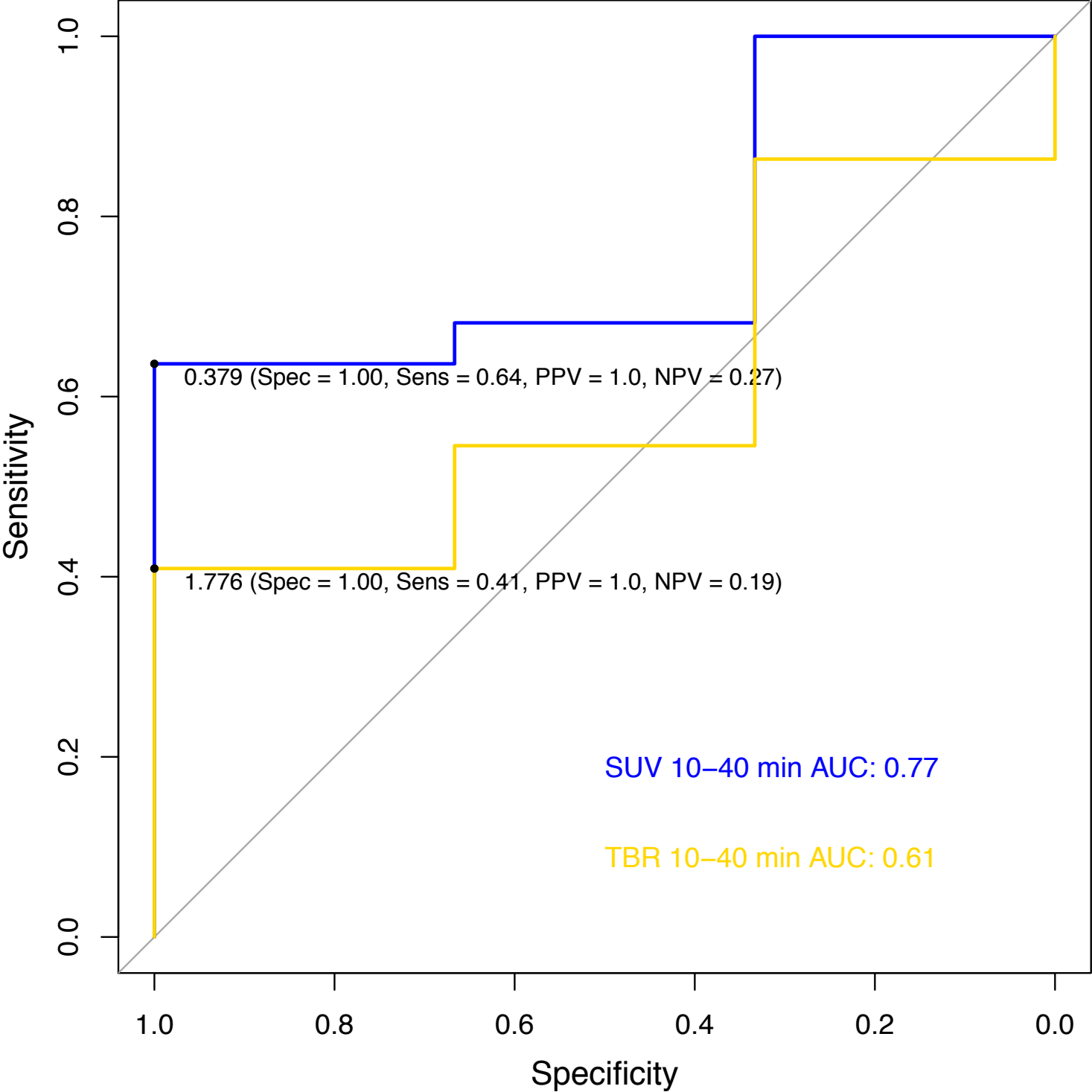

Supplement: Supplementary file 3 — Comparison of [11C] choline SUV and TBR in low-grade gliomas. (PDF 96 kb) [file 13550_2019_523_MOESM3_ESM.pdf]

## Comparison of [18F]FET SUV and TBR in high-grade gliomas

A)

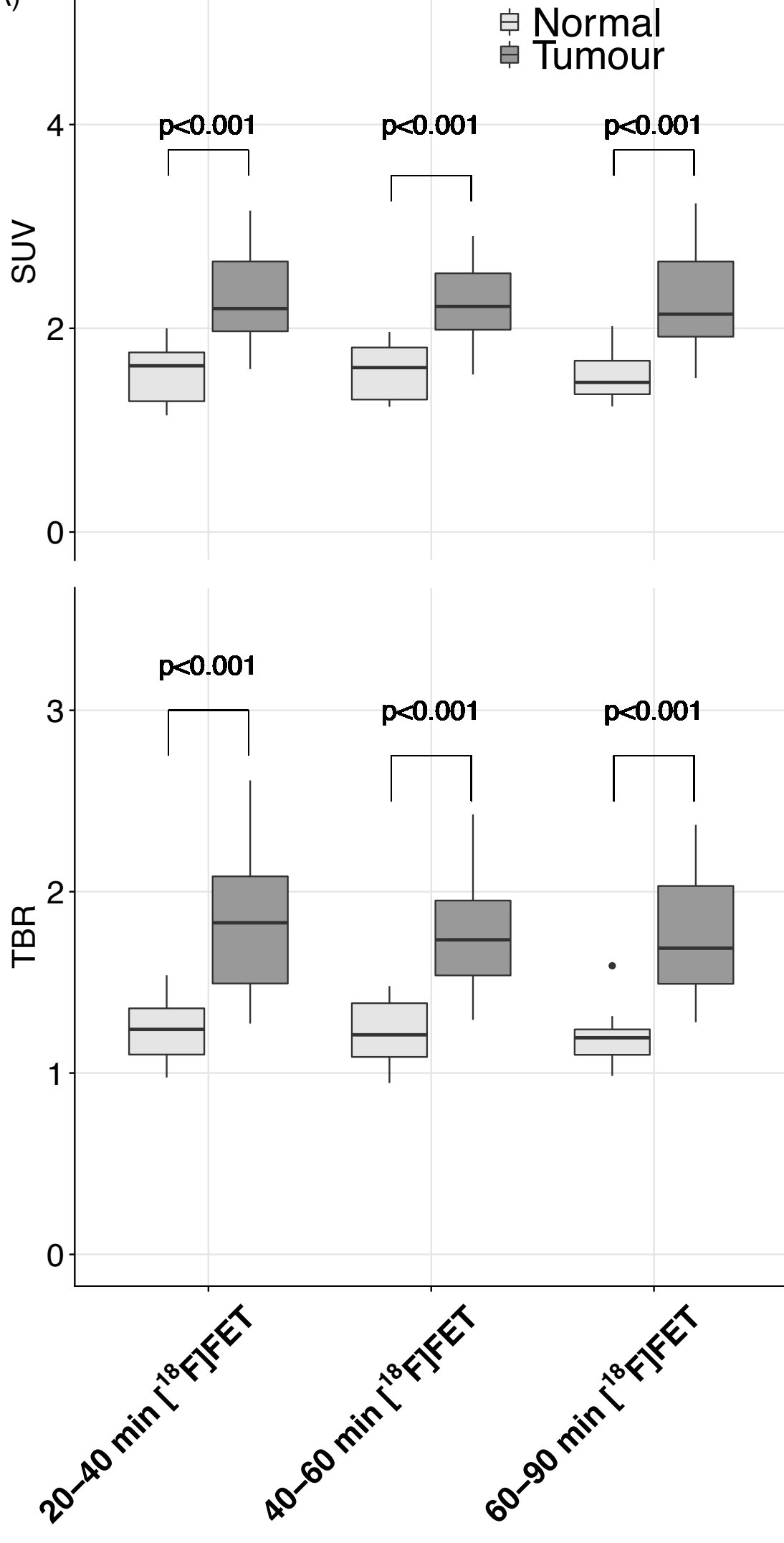

B)

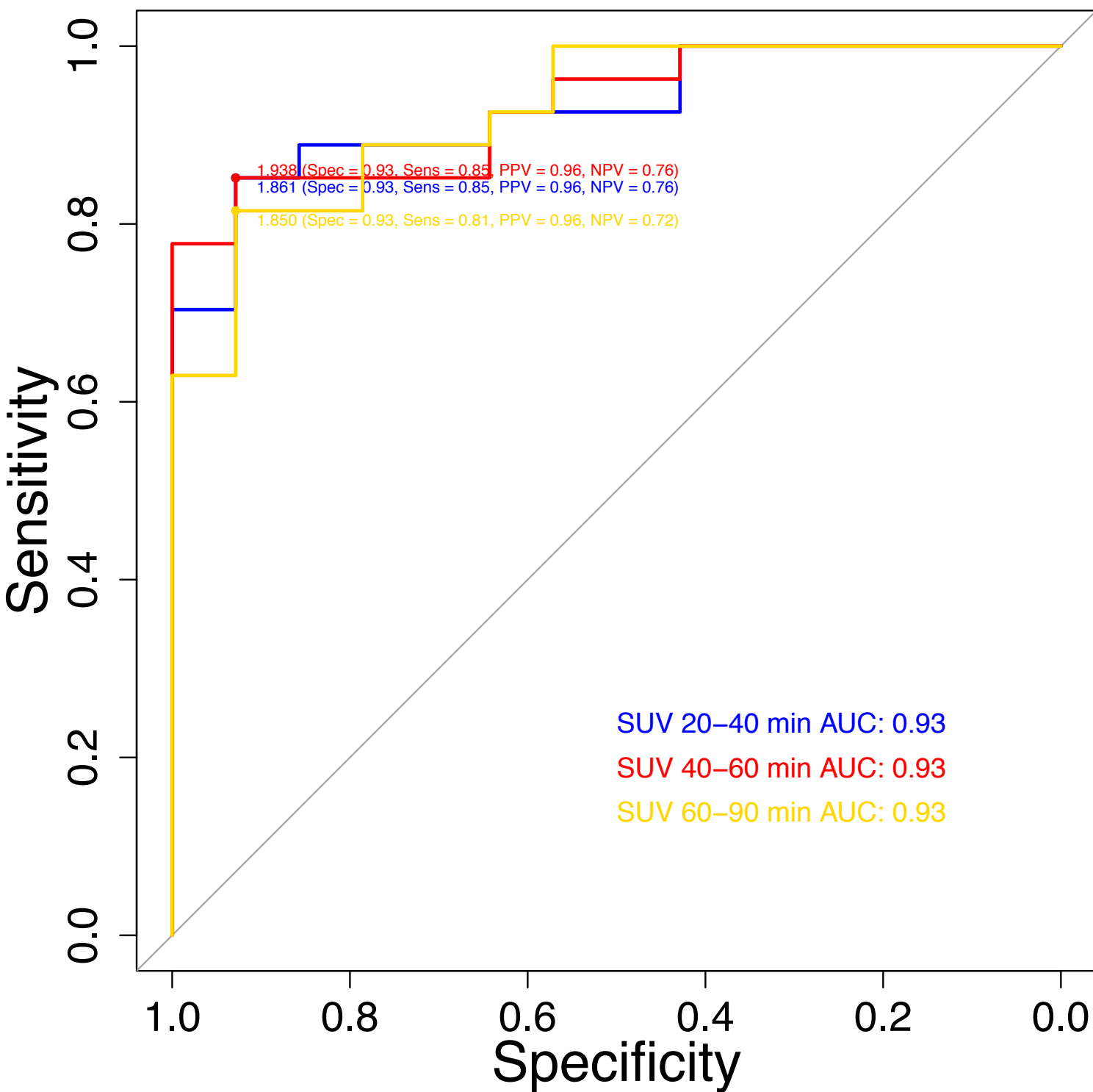

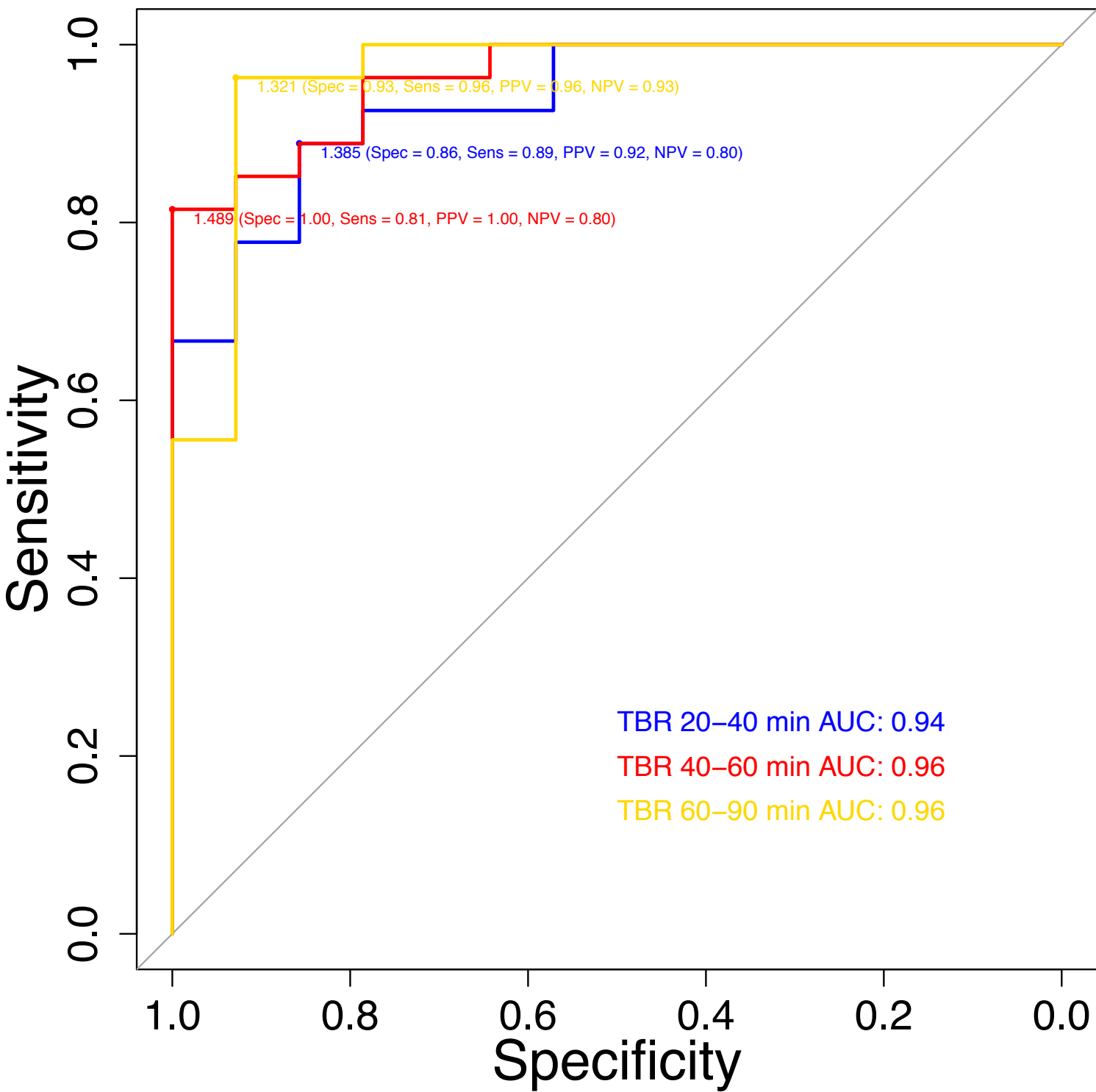

Supplement: Supplementary file 4 — Comparison of [18F] FET SUV and TBR in high-grade gliomas. (PDF 111 kb) [file 13550_2019_523_MOESM4_ESM.pdf]

Online Resource 5: Comparison of [18F]FET SUV and TBR in low-grade gliomas

A)

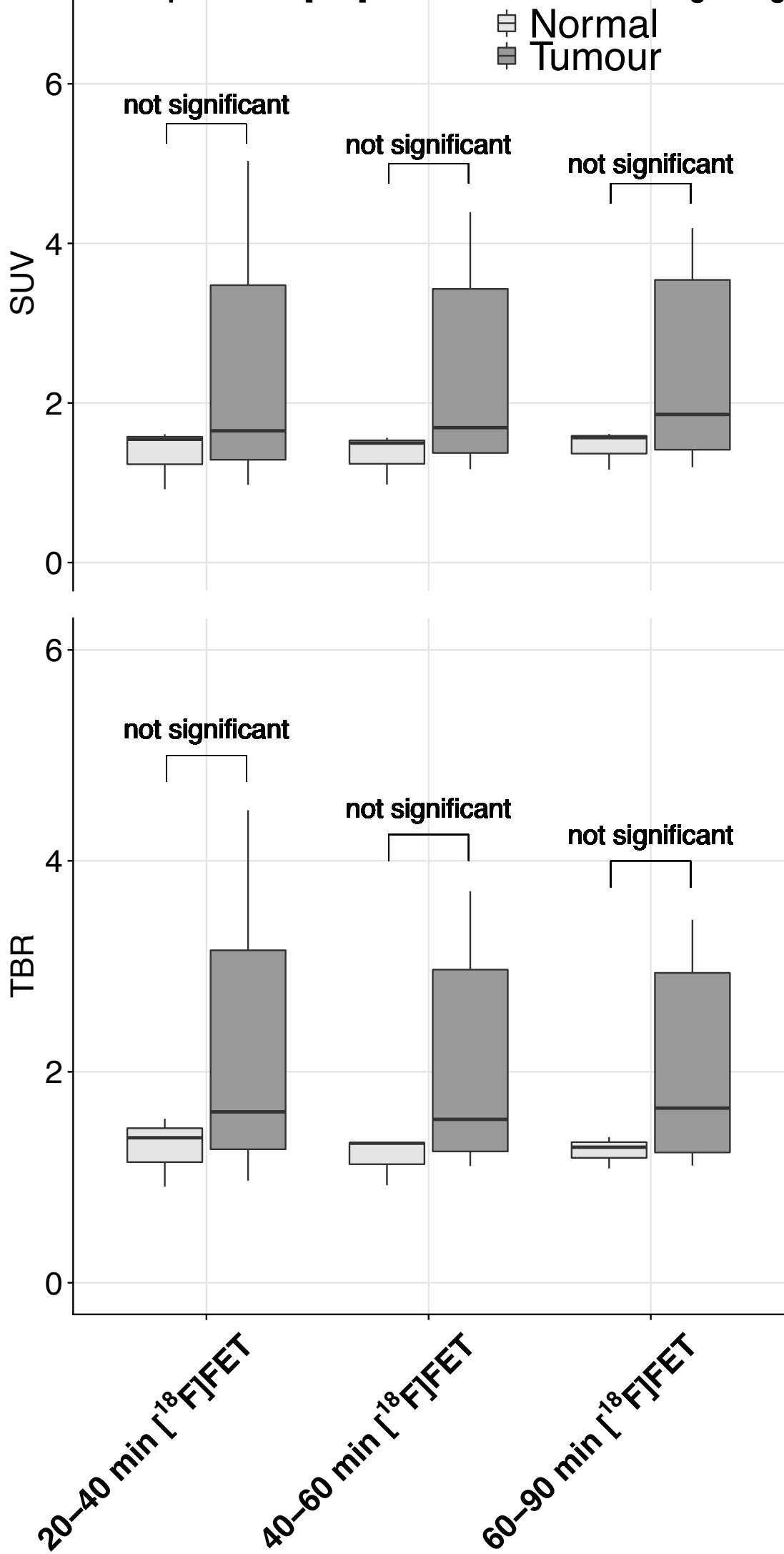

B)

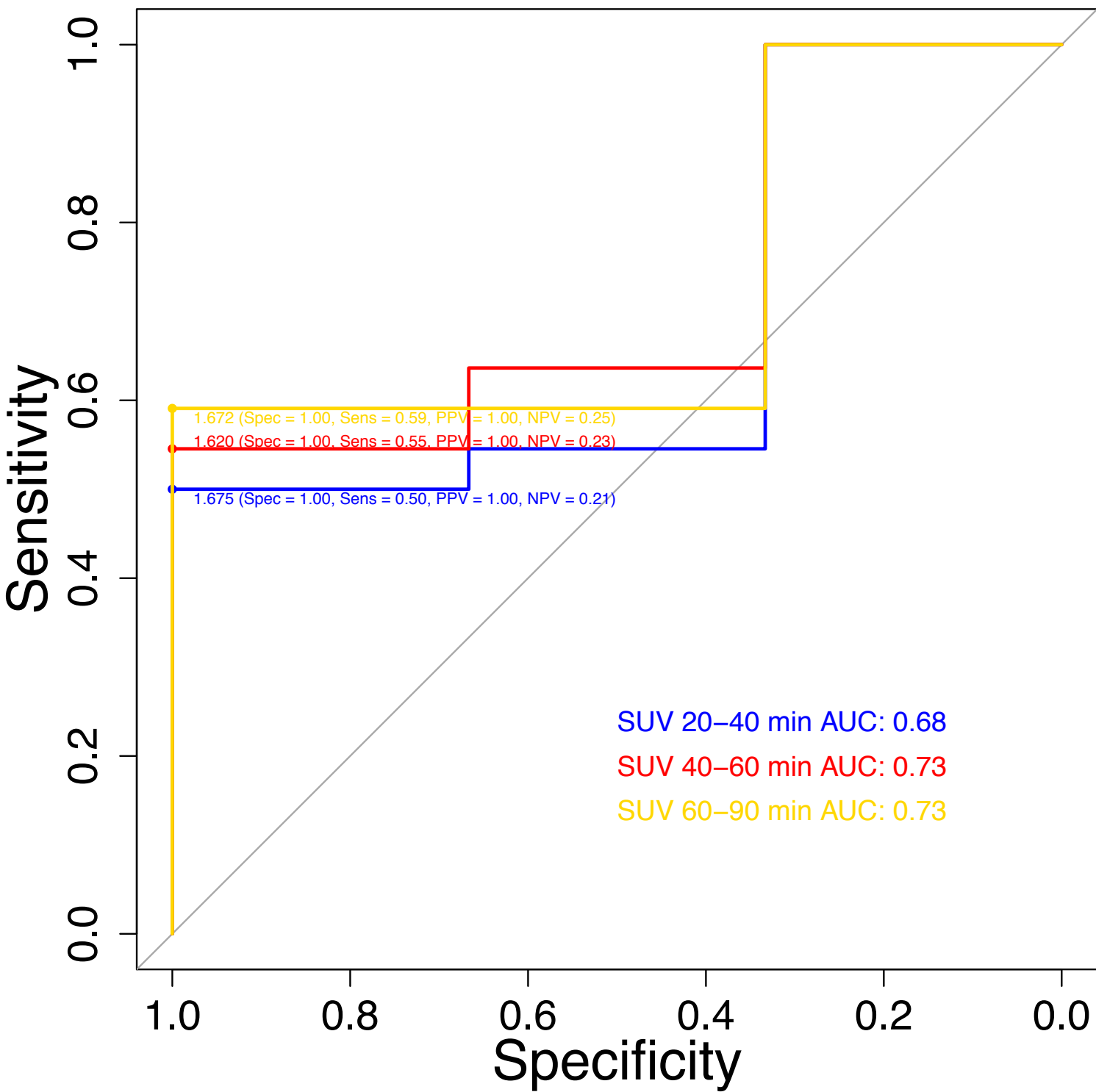

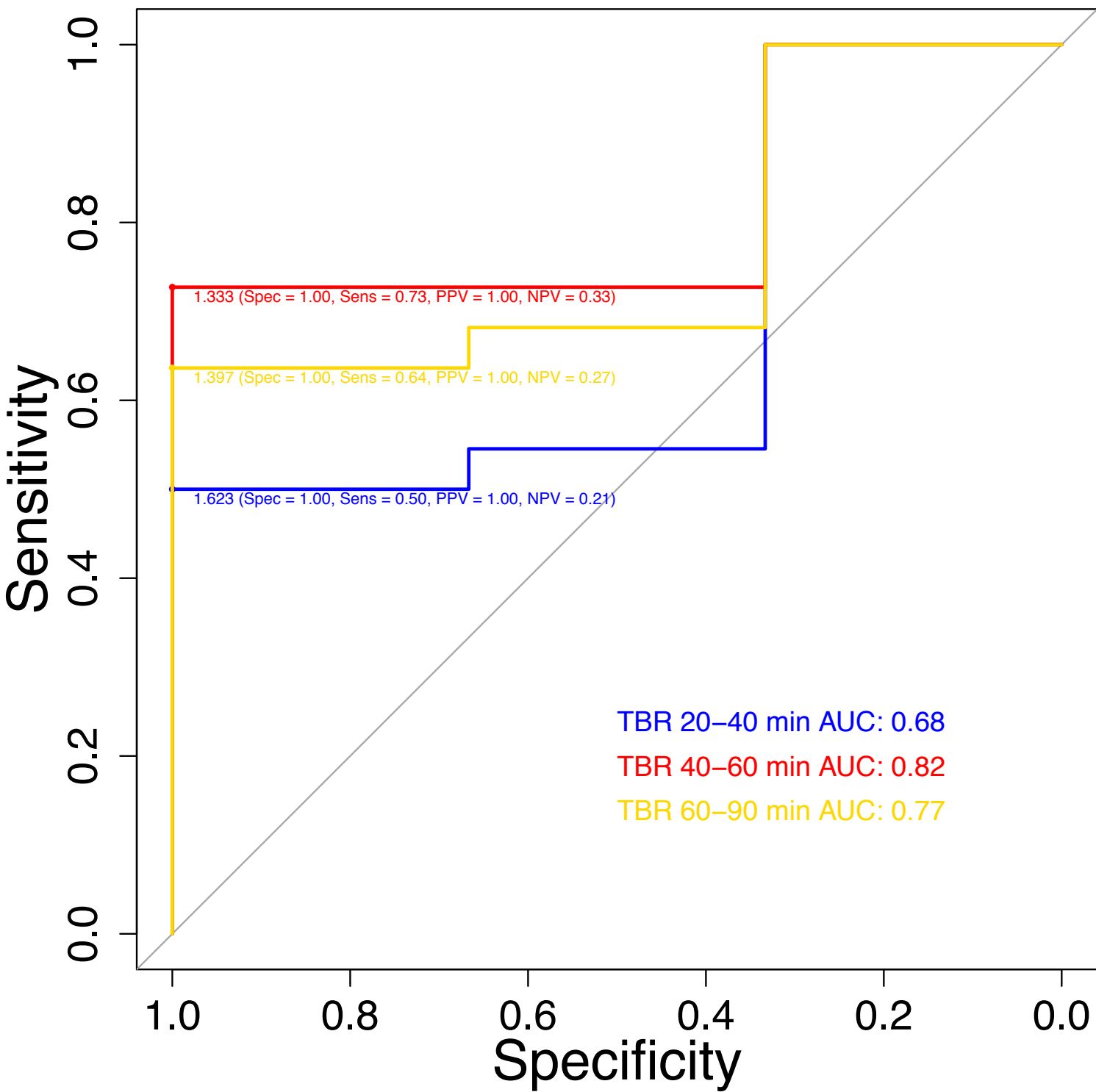

Supplement: Supplementary file 5 — Comparison of [18F] FET SUV and TBR in low-grade gliomas. (PDF 108 kb) [file 13550_2019_523_MOESM5_ESM.pdf]

[<sup>18</sup>F]FET

[<sup>11</sup>C]choline

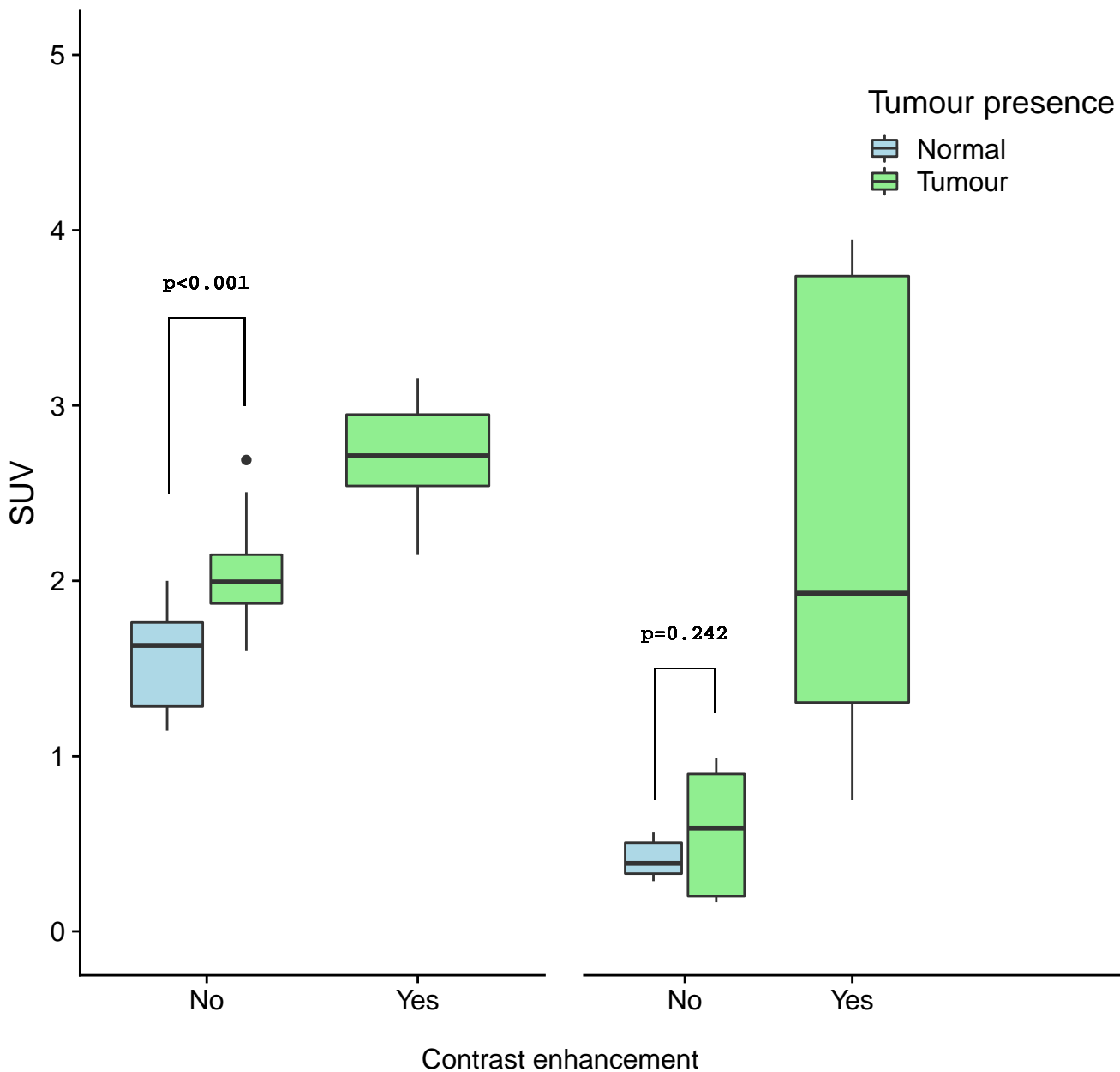

Supplement: Supplementary file 8 — Boxplot of [11C] choline and [18F] FET SUV in samples with and without contrast enhancement in enhancing gliomas. (PDF 5 kb) [file 13550_2019_523_MOESM8_ESM.pdf]
