# Supplementary material for: Direct comparison of [11C] choline and [18F] FET PET to detect glioma infiltration: a diagnostic accuracy study in eight patients
Source: EJNMMI Res. 2019 Jun 28;9:57. doi: 10.1186/s13550-019-0523-8 (PMC6598977; doi:10.1186/s13550-019-0523-8)
Supplement: Supplementary file 6 — ROC curve of [11C] choline and [18F] FET in high- and low-grade gliomas. (PDF 66 kb) [file 13550_2019_523_MOESM6_ESM.pdf]

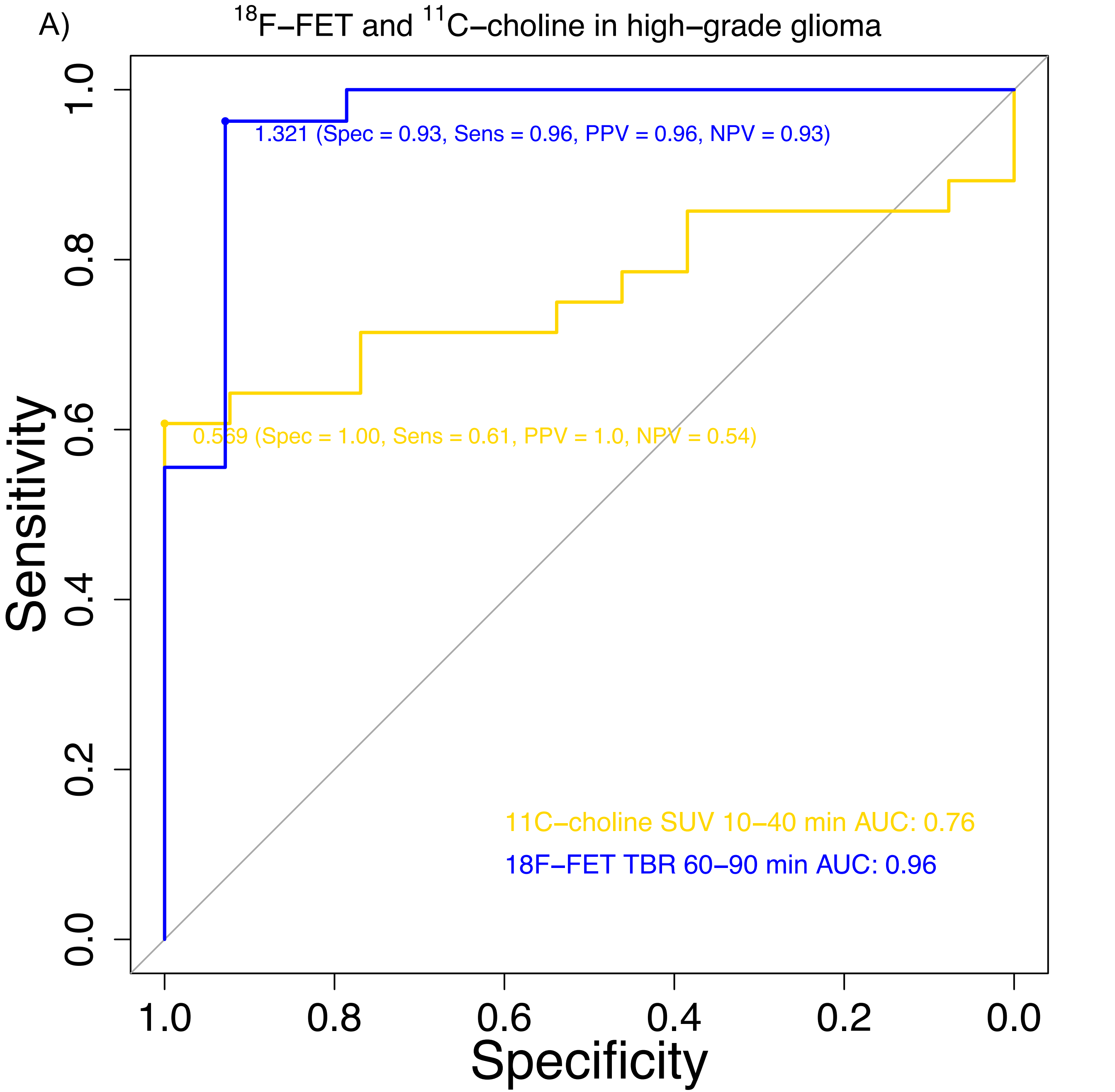
A)
18F–FET and 11C–choline in high–grade glioma
1.321 (Spec = 0.93, Sens = 0.96, PPV = 0.96, NPV = 0.93)
0.569 (Spec = 1.00, Sens = 0.61, PPV = 1.0, NPV = 0.54)
11C–choline SUV 10–40 min AUC: 0.76
18F–FET TBR 60–90 min AUC: 0.96
Sensitivity
Specificity
0.0
0.2
0.4
0.6
0.8
1.0

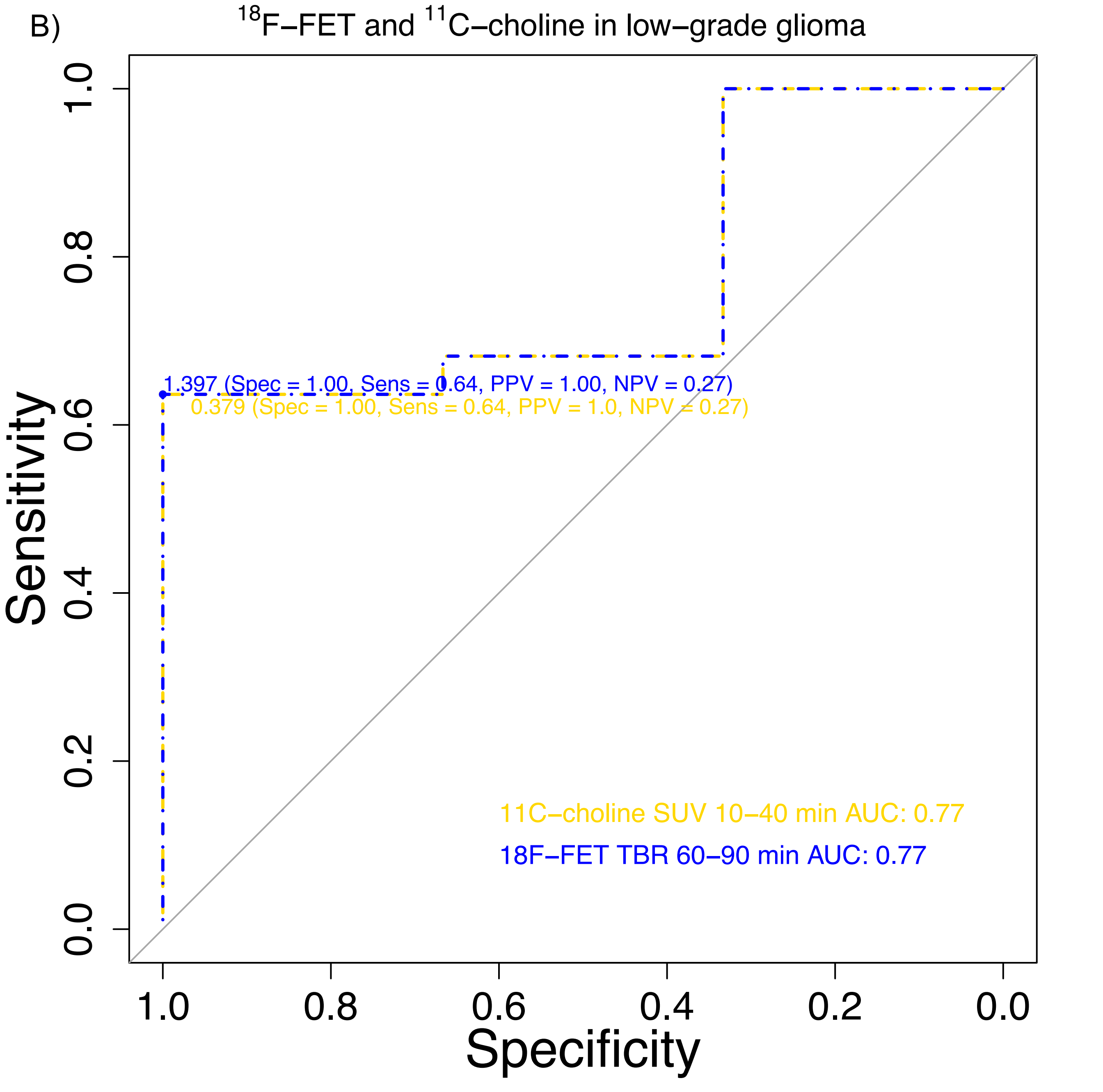
B)
18F–FET and 11C–choline in low–grade glioma
1.397 (Spec = 1.00, Sens = 0.64, PPV = 1.00, NPV = 0.27)
0.379 (Spec = 1.00, Sens = 0.64, PPV = 1.0, NPV = 0.27)
11C–choline SUV 10–40 min AUC: 0.77
18F–FET TBR 60–90 min AUC: 0.77
Sensitivity
Specificity
1.0
0.8
0.6
0.4
0.2
0.0
